# Supplementary material for: A Combination of Transcriptome and Enzyme Activity Analysis Unveils Key Genes and Patterns of Corncob Lignocellulose Degradation by Auricularia heimuer under Cultivation Conditions
Source: J Fungi (Basel). 2024 Aug 3;10(8):545. doi: 10.3390/jof10080545 (PMC11355066; doi:10.3390/jof10080545)
Supplement: Supplementary file 1 [file jof-10-00545-s001.zip › Supplementary Figures.pdf]

Supplementary Figure S1

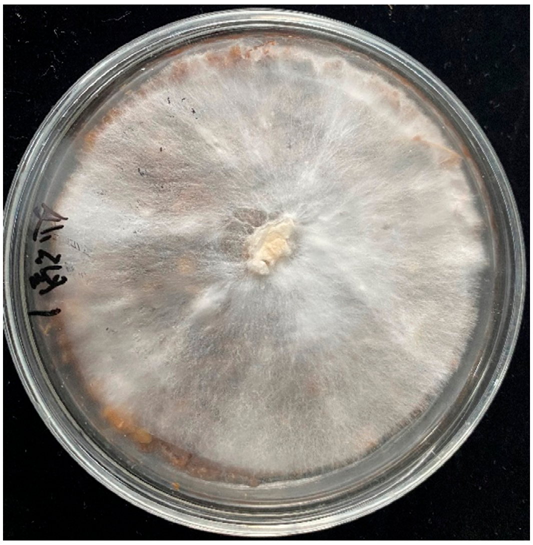

The mycelia of treatment 1

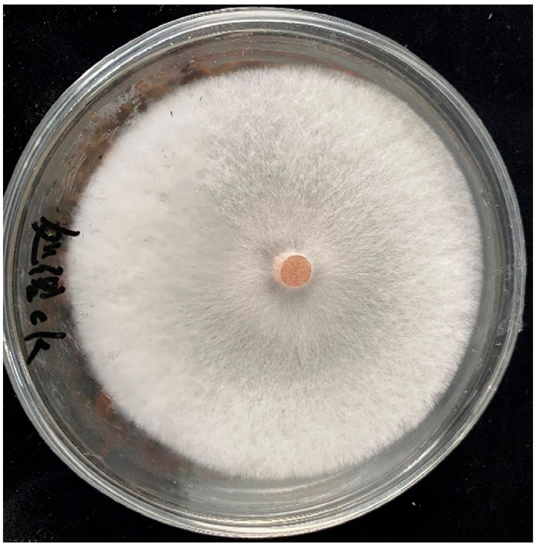

The mycelia of CK

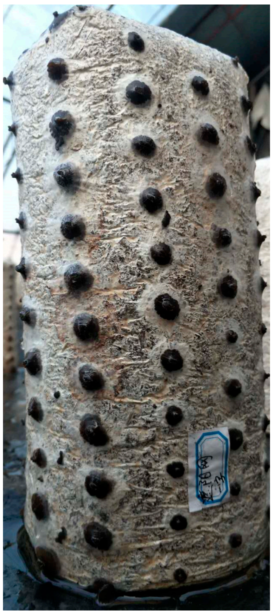

The mycelia of treatment 1

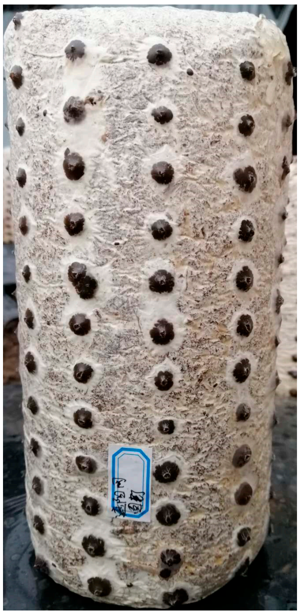

The primordia of CK

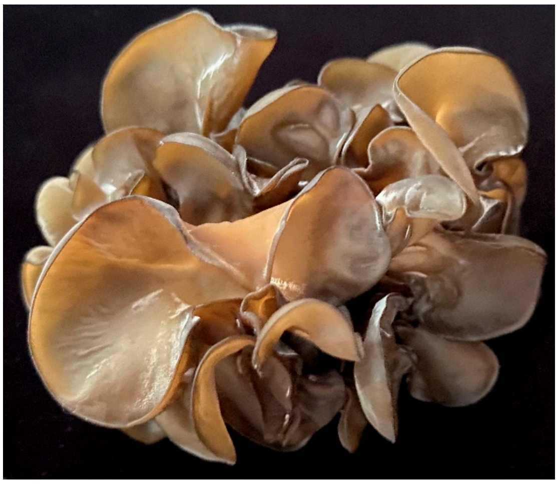

The fruiting body of CK

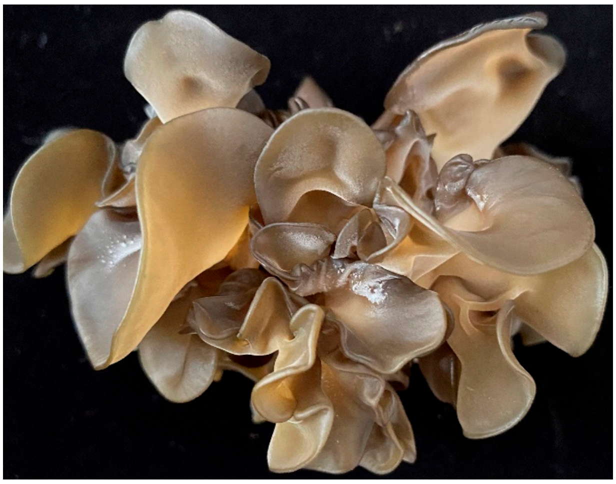

The fruiting body of treatment 1

Figure S1. The mycelia, primordia and fruiting bodies of treatment 1 and CK.

Supplementary Figure S2

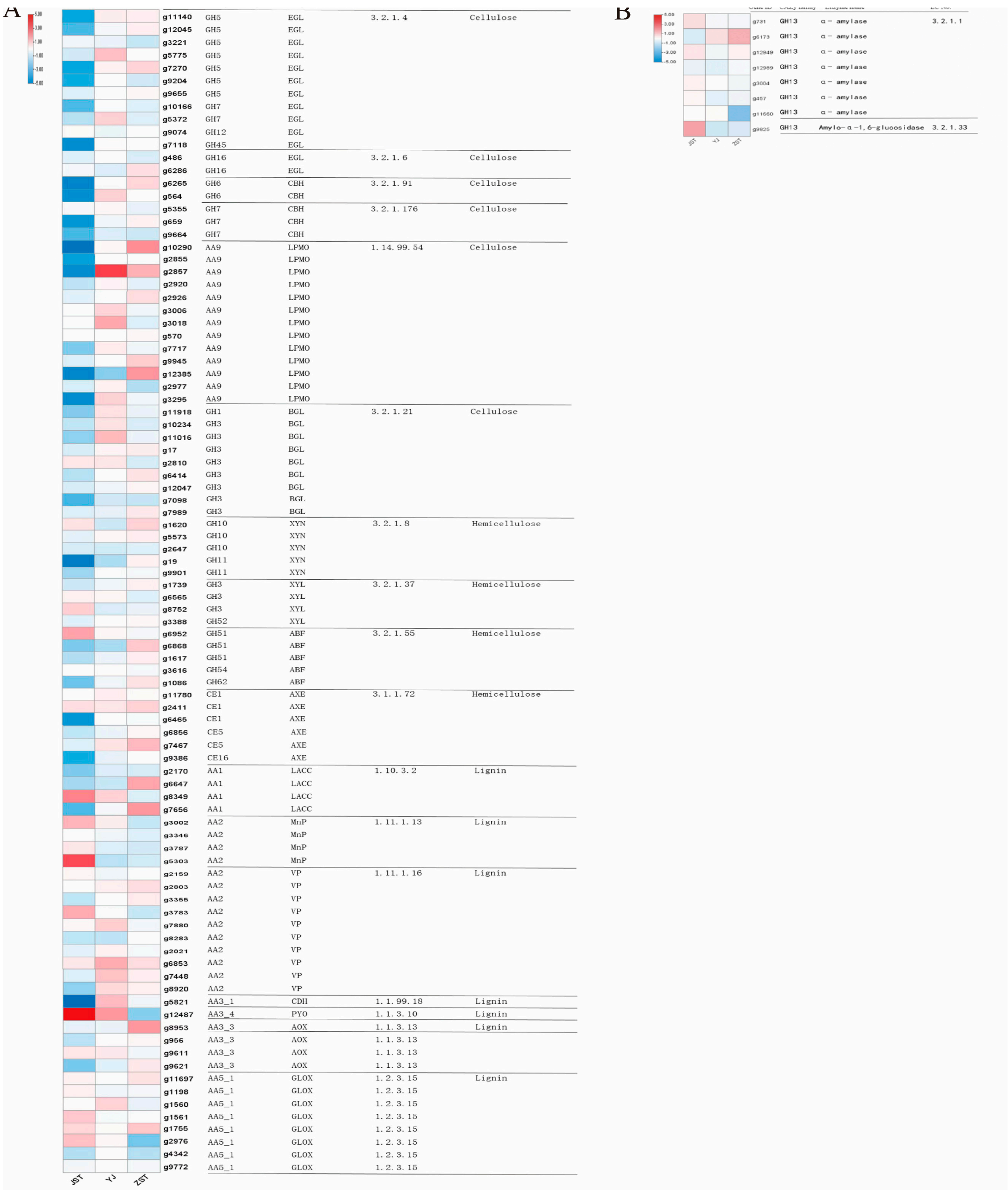

Figure S2. The CAZyme DEGs of transcriptomes from T1. Lignocellulose decomposition(A) and starch decomposition(B). The red and blue represented the up-regulated DEGs and the down-regulated DEGs respectively.

Supplementary Figure S3

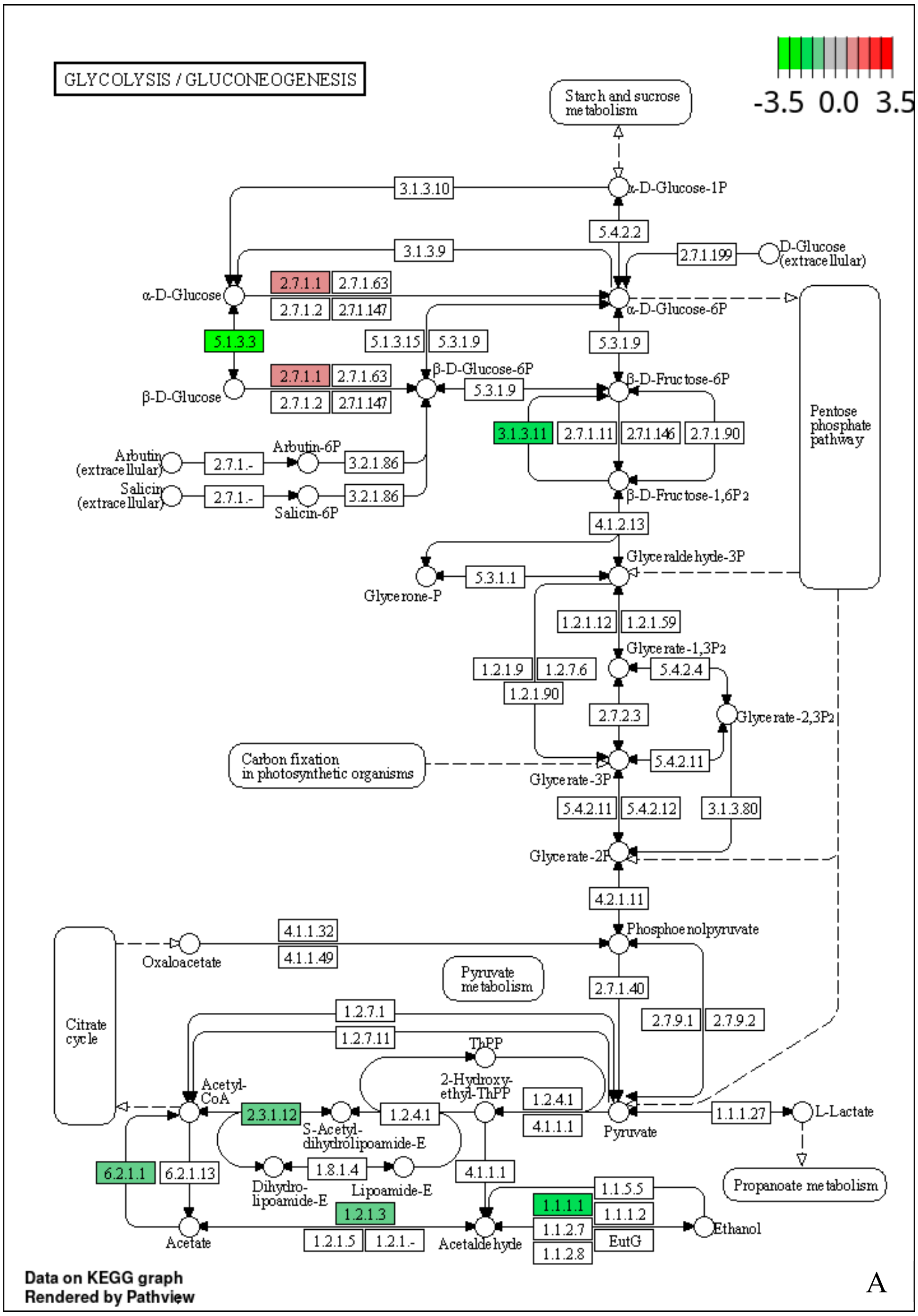

A

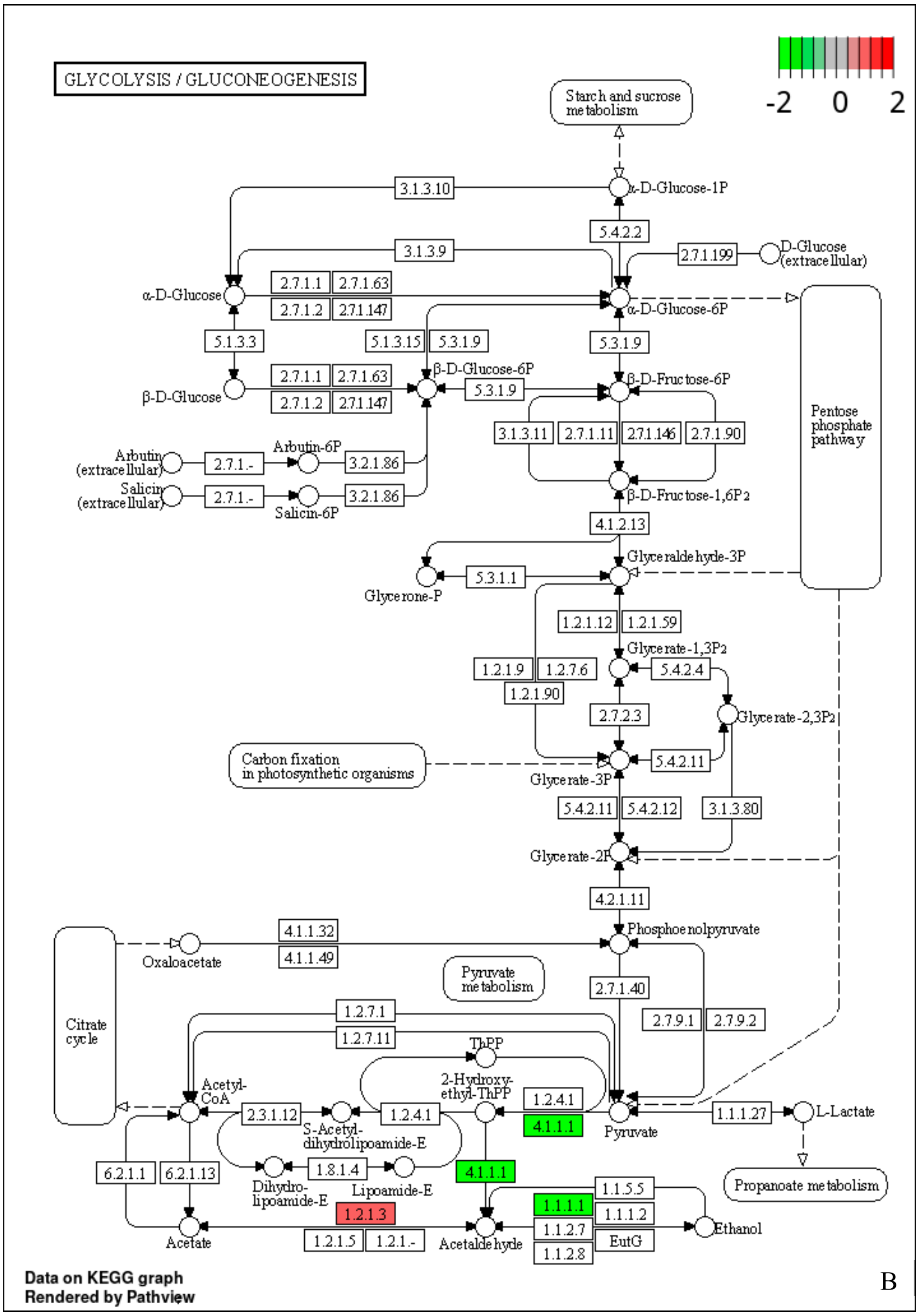

Figure. S3. The glycolysis/gluconeogenesis pathway(adl00010) of T1. A: The mycelial stage; B: The fruiting stage. The number in the frame represents the enzyme number, which is stipulated by the Enzyme Commission. The red and green frames indicate that the enriched genes are upregulated or downregulated. The circle represents the metabolite, and the arrow represents the enzymatic reaction.

Supplementary Figure S4

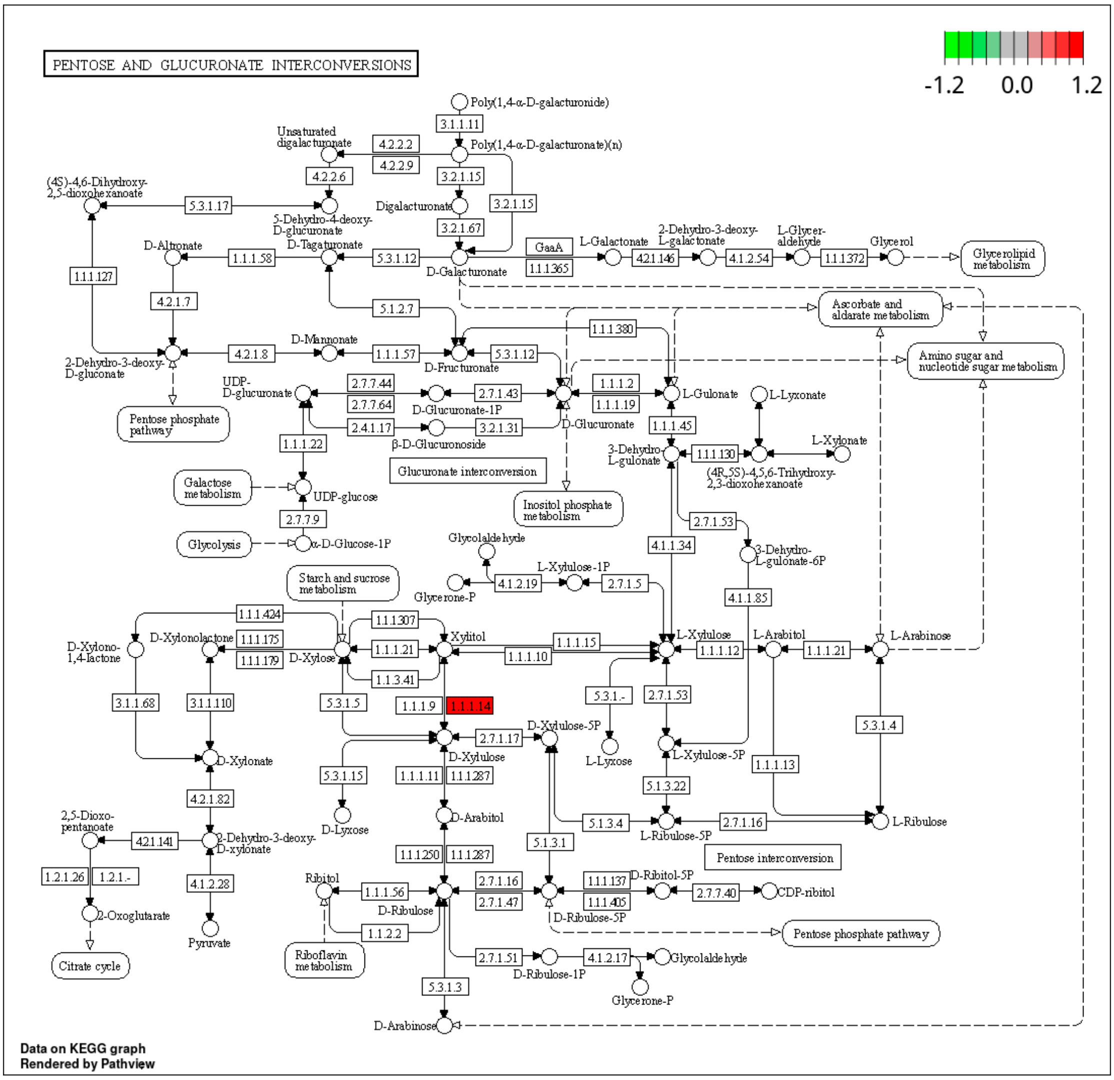

Figure. S4. Pentose and glucuronate interconversions pathway(adl00040) of T1 at the fruiting stage. The number in the frame represents the enzyme number, which is stipulated by the Enzyme Commission. The red and green frames indicate that the enriched genes are upregulated or downregulated. The circle represents the metabolite, and the arrow represents the enzymatic reaction.

Supplementary Figure S5

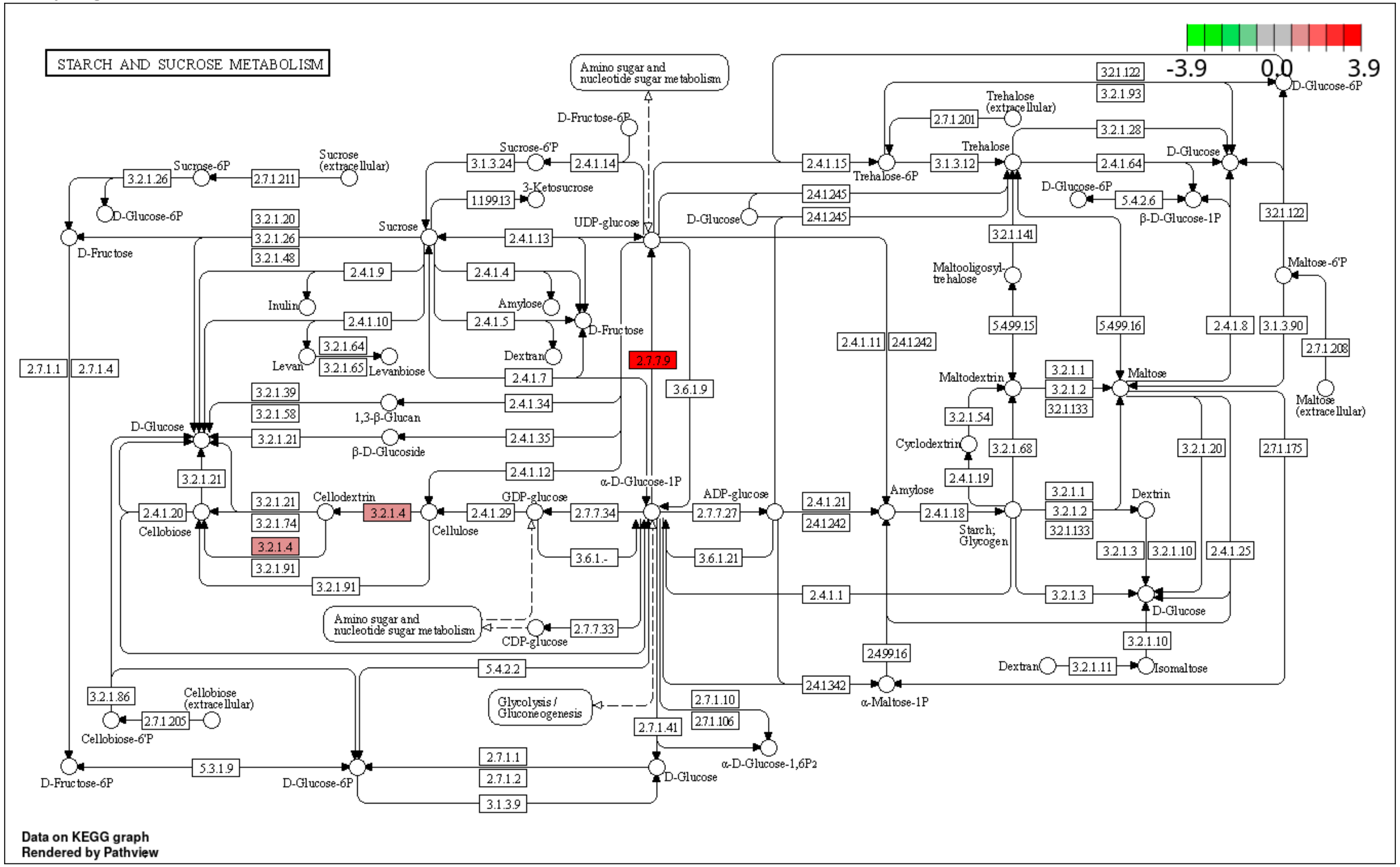

Figure. S5. The Starch and sucrose metabolism pathway(adl00500) of T1 at the primordia stage.

The number in the frame represents the enzyme number, which is stipulated by the Enzyme Commission. The red and green frames indicate that the enriched genes are upregulated or downregulated. The circle represents the metabolite, and the arrow represents the enzymatic reaction.
